# The Four Dangers

**Published:** 1873-09

**Authors:** 


### THE FOUR DANGERS.

THE leading nations of antiquity rose to wealth, power and renown, and then descended—to the dogs. They fizzled out, every one of them; instead of being a success, they, one and all, came out at the little end of the horn. The Greeks, with all their culture, their poetry, their music, their philosophy, fell into insignificance; and Rome, with her military *prestige*, her athletic games, her baths, her physical training, her oratory and her world-wide conquests, ended her career in effeminancy, extravagance and gluttony. The greatest families of the greatest nations die out in a few generations—and so with individuals; they rise to a certain height in wealth, influence or renown, and sooner or later decadence comes to all of them. Can it be that every nation must have within itself the seeds of death, as the individual does? The Church, the kingdom of God, has within itself a well of water, springing up unto everlasting life. It must "increase," until it shall pervade all lands. May not a nation become a Christian nation and live forever, or at least until that eventful hour when the angel shall announce that

"TIME SHALL BE NO LONGER."

The English nation, which had in it most of the Christian element, is the greatest on the globe. But to come nearer home, has our nation within itself the seeds of death, or has it so much of the "living wine" of the Church that, with the Church, it will live until the consummation of all things? A man may have within himself some element of disease that will eventuate in death, but which may, by the skill of the physician, be removed from the system. So may it be with a nation. The seeds of death may be sown in it, or these seeds may be removed. As to the greatest dangers of the United States, different persons may and do have different views; it may be a public benefit to compare notes, and if it should lead to thoughtfulness first, and to practical action in the near future, then there is hope ahead. Men may say what they please, but one great fact shines out, like a sun in a midnight sky—the Christian religion is

THE SALT OF THE EARTH.

It is as well the salt (the preservative element) of all nations—of any nation—and, without these elements largely permeating the sentiments of the people, this glorious country of ours will make shipwreck on the rock of

INFIDEL ANARCHY.

People may talk as they please about the school house and the college; about civilization and culture; about music, and poetry, and painting, and all that; if every man, woman and child was a Raphael, a Euclid, a Porson, a Bacon, a Lynd, a Powers, a Handel, all combined, without the religious element, it would not be ten years before the United States would be a model Pandemonium; for the greater the culture the greater the capacity for demon deeds. The things which, to our view, are sapping out our national life-blood, are loose views about Sunday, the Bible, divorce, clerical support.

If you give up Sunday to purposes of recreation and pleasure, you give up the Christian religion, and whatever we do to present a motive for keeping the people from going to church on Sundays, is but the entering wedge to our national undoing as a free and happy people. Every public park, every Sunday excursion, every library opened, every "sacred concert" given—these are but downward steps. Whatever inducement is presented to young or old to neglect public worship, to neglect going to meeting on Sundays, is a public danger. When a great man comes to New York for the first time we give him a public reception; men leave their business, encounter expense, subject themselves to inconvenience in order to pay their respects to the person whom they seek to honor, and thus they show to the people their appreciation of his character and principles. So does going to meeting on Sundays give men an opportunity of showing to all that they appreciate the principles of the Christian religion, and that they want to hear of God. Surely it is wrong to yield to inducements to prevent such an honoring; wrong to offer such inducements to others. Shall we pay our respects publicly to a man, and not to our Maker, on the day which He has appointed for that express purpose?

